# Outcomes of Ilizarov ring fixation in recalcitrant infected tibial non-unions – a prospective study

**DOI:** 10.1186/1752-2897-2-6

**Published:** 2008-07-23

**Authors:** Thayur R Madhusudhan, Balasundaram Ramesh, KS Manjunath, Harshad M Shah, Dabir C Sundaresh, N Krishnappa

**Affiliations:** 1 Trauma and Orthopaedics, Glan Clwyd Hospital, Rhyl, UK; 2 Consultant Orthopaedic Surgeon, Glan Clwyd Hospital, Rhyl, UK; 3 Trauma and Orthopaedics, Bangalore Medical College, Bangalore, India; 4 Trauma and Orthopaedics, M S Ramaiah Medical College, Bangalore, India

## Abstract

**Background:**

Infected non-union of long bones is a problem in the developing countries. Persistent infection, deformity, shortening, bone loss, joint stiffness and disability complicate the non-union. Secondary procedures are often required for correction of bone defects and deformity. Ilizarov method addresses all the above problems simultaneously and offers a panacea for infected non-unions. The stability of the fixation and provision for bone transport allows bridging of bone defects, limb lengthening, early weight bearing ambulation and joint mobilisation.

**Aim of the study:**

To know the suitability of this procedure in recalcitrant infected tibial non-unions in the Indian population and the influence of socio-economic factors in the functional outcome.

**Method of study:**

This was a 3-year prospective study in 22 consecutive patients with an average follow up of 13 months following fracture union. The results were analysed using the ASAMI scoring system.

**Results:**

Of 22 patients in the study, 13 patients who underwent external bone transport, had an average bone gap of 4 cms [2–11 cms] with an average duration of fixation of 9.3 months [6.5–13 months]. There were 4 excellent, 3 good, 4 fair and 2 poor bony results and 1 excellent, 3 good, 6 fair and 2 poor results. 1 patient was lost for follow up at final functional analysis. 9 patients who underwent internal bone transport had an average bone gap of 5.4 cms [1.5–9 cms] with an average duration of fixation of 8.5 months [4–11 months]. There were 3 good 4 fair and 2 poor bony results and 1 good, 3 fair, and 2 poor functional results. Good to excellent results were witnessed in well-motivated patients with adequate social and financial support. Patients with fair to poor results preferred amputation to limb salvage despite the fact that they retained their limbs.

**Conclusion:**

Treatment of infected non-unions of Tibia with Ilizarov ring fixation is effective but for optimal results the treatment needs to be individualised by the treating surgeon with due consideration of the socio-economic factors.

## Background

Infected non-union of long bones is a problem in the developing countries. Persistent infection, deformity, shortening, bone loss, joint stiffness and disability complicate the non-union. Conventional treatment including extensive debridements and coverage of tissue defects with flaps or skin grafts, antibiotic bead packing of the defects, papineau open cancellous grafting, tibio fibular synostosis, free tissue transfer including bone transplants, address the problem of infection and non union primarily. Secondary procedures are often required for correction of bone defects and deformity. This eventually results in multiple surgeries and scarring of the tissues with joint stiffness and oedema, which interfere with an optimal limb function.

Ilizarov method addresses all the above problems simultaneously and offers a panacea for infected non-unions. The stability of the fixation allows weight bearing ambulation and joint mobilisation. Progressive bone histogenesis following corticotomy and bone transport helps in filling bone gaps eradicating infection and promoting fracture union. Infection control is achieved by radical debridement of the infected tissues including bone and followed by bone transport to reconstruct the residual bone defects.

We present our experience of Ilizarov fixation in the treatment of established recalcitrant infected non-unions of the tibia and the suitability of this procedure in the Indian population with particular reference to bony union and identify the factors influencing the functional outcomes.

## Methods

32 patients with established infected non-union of the tibia were initially considered for the study.4 patients rejected the fixator at the initial stage and preferred amputation. 4 patients after initial acceptance opted out of the study and 2 patients failed to report back after initial acceptance. The rest 22 patients were included for Ilizarov ring fixation over a 3 year period prospectively.

Clinical history including co-morbidities, social habits including smoking and alcohol consumption, previous treatment offered for the fracture, complications, duration of nonunion, time of referral and occupation. Socio-economic and educational status of the patients were documented. 12 patients were agriculturists and the rest were employed in government and private sector organisations. 13 patients belonged to the middle class and 9 in the lower class [1]. All patients were active and sole earners of their families with no additional source of income.

All patients had preoperative full-length radiographs of the affected leg for assessment of the level and type of fracture nonunion, plane of deformity, bone quality and presence of sequestrum. All patients were counseled about the procedure to be performed, and the expected outcome of treatment. All patients were optimised preoperatively for the proposed operation. Physiotherapy within comfort with specific reference to joint mobilisation and oedema control was attempted in all patients. Culture swabs from draining sinuses and open wounds were carried out in all patients and appropriate antibiotic therapy was initiated. This was repeated whenever necessary throughout the duration of treatment.

The site of non-union was in the distal, middle and proximal thirds in 12, 7 and 3 patients respectively. The initial diagnosis was Gustilo type 1 open fractures in 1, Type 2 in 6 and type 3 in 14 patients [2]. 1 patient had closed fracture treated with internal fixation. 3 patients had extensive bone loss at the time of initial injury. There were 25 debridement procedures in total, 11 patients had internal fixation device [Intra-medullary nail in 7 and Plate and screw fixation in 4] and, 11 had external fixation followed by plaster immobilisation as definitive treatment. 4 patients with internal fixation had had fascio-cutaneous flap coverage for the open wounds. The number of procedures per patient ranged from 2–5 [Average 3]. The co morbidities were Diabetes in 2 patients, Hypertension in 1 patient, chronic obstructive pulmonary disease in 2 patients. There was history of smoking and alcohol consumption in 18 patients. 2 patients were on treatment for depressive illness.

The average duration of non-union and time of referral to our centre was 7.8 months. Limb shortening ranged from 3 – 11 cms and bone gap ranged from 2–9 cms. 13 patients had associated fibular shaft fractures, which had healed at the time of presentation. Pus culture in all patients obtained pre operatively, revealed a mixed a bacterial growth.

The Ilizarov frame was constructed pre-operatively in 18 patients and intra-operatively in 4 patients. 9 patients had wound debridements and sequestrectomy prior to ring fixator application. The limb was supported in a plaster slab and elevated until ring fixation in the interval period. 13 patients had debridements combined with ring fixator application as a single stage procedure. All patients had bifocal osteosynthesis [compression of the fracture site with bone transport following corticotomy]. 13 patients with bone loss and limb shortening had external bone transport [Acute docking of the fracture site with/without fibular osteotomy followed by gradual distraction at the corticotomy site]. 11 of these had acute docking and two patients with large bone gaps had gradual bone transport. 9 with bone loss but no limb shortening had had internal bone transport [Bone transport with gradual distraction at the corticotomy site with an intact fibula]. 13 patients had proximal tibial and 9 patients had distal tibial corticotomies. Postoperatively all patients had radiographs of tibia and fibula for assessment of the corticotomy and position of the wires. Corticotomy site distraction was initiated between 5–7 days at the rate of 1 mm per day and compression and distraction technique [Accordian manoeuvre] was employed in 2 patients. Follow up x-rays were done at 3 weeks for assessment of the regenerate and at 4 weeks interval thereafter until fracture union. In doubtful cases ultrasound assessment of the regenerate was performed and distraction rate was reduced to 0.5 mm/day until satisfactory appearance on x-rays.

Patients were mobilised partial weight bearing, within comfort by a trained physiotherapist. Patients were discharged upon satisfactory compliance and followed up in the fracture clinics at monthly intervals for assessment of fracture union, regenerate progress and ensuring compliance with physiotherapy. Fixator was retained further for the duration equal to the period of bone transport after bone docking. Bone union was confirmed by conventional x-rays and the fixator was removed under anaesthesia. The operated limb was protected in a functional cast brace for at-least twice the duration of bone transport.

The period of follow up after fracture union ranged from 6 – 20 months [Average 13 months]. 4 patients rejected the fixator during the course of treatment and follow-up. The outcomes were assessed using the Association for the Study and Application of Methodology of Ilizarov [ASAMI] criteria.

## Results

**Figure 1:**
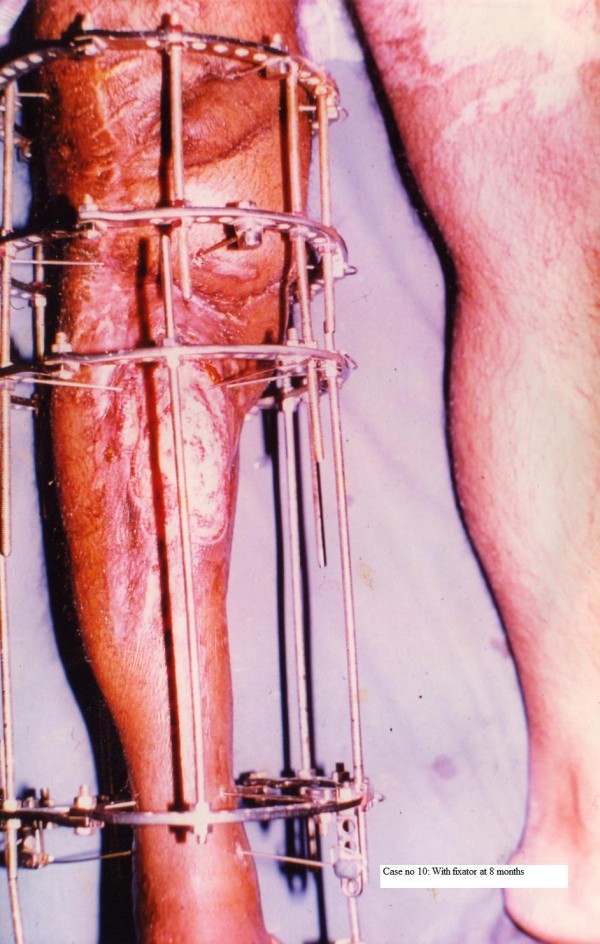
Patient No 10: with fixator at 8 months.

**Figure 2:**
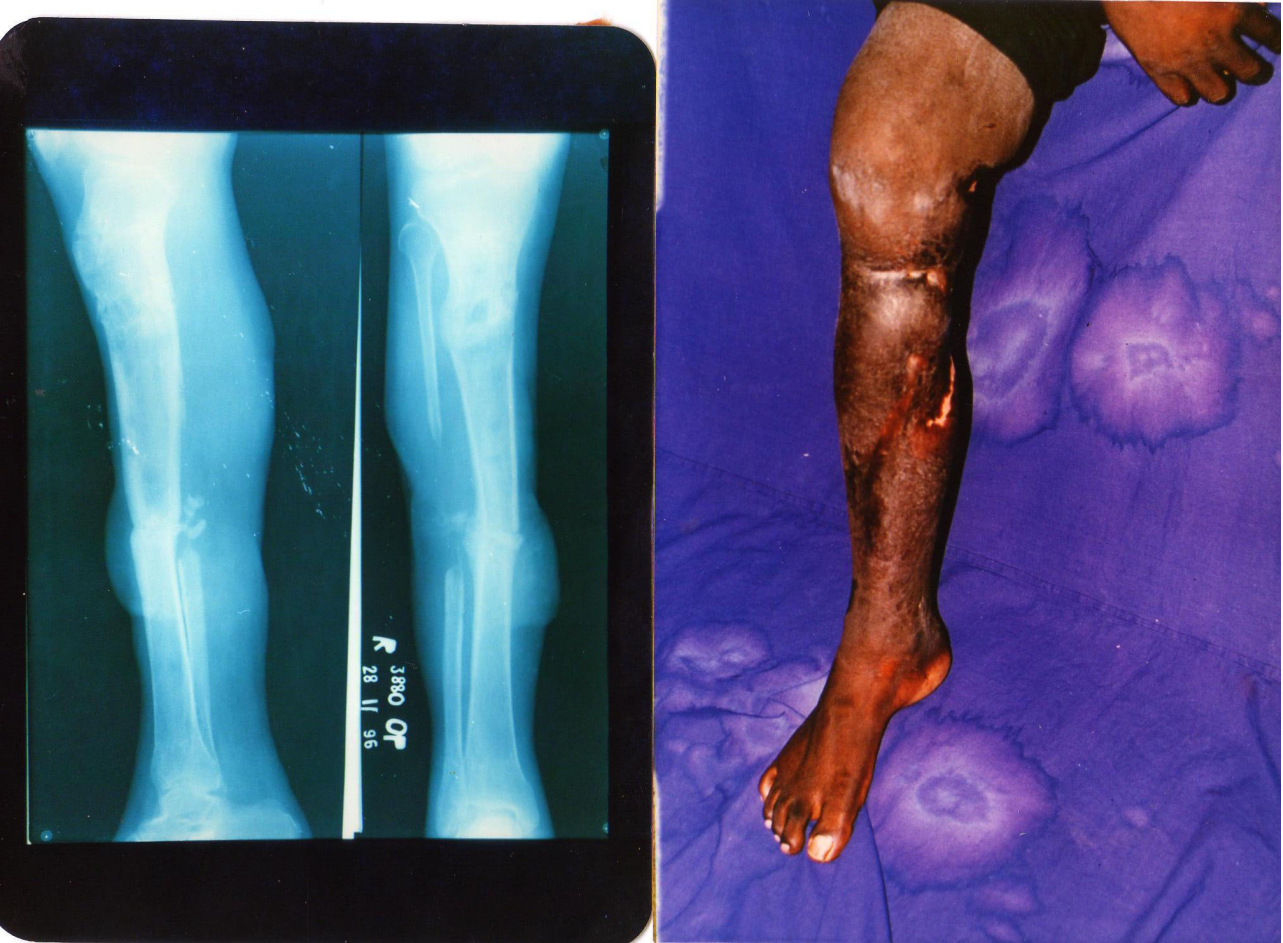
Patient no 10: follow up x-ray and clinical photograph at 12 months.

**Figure 3:**
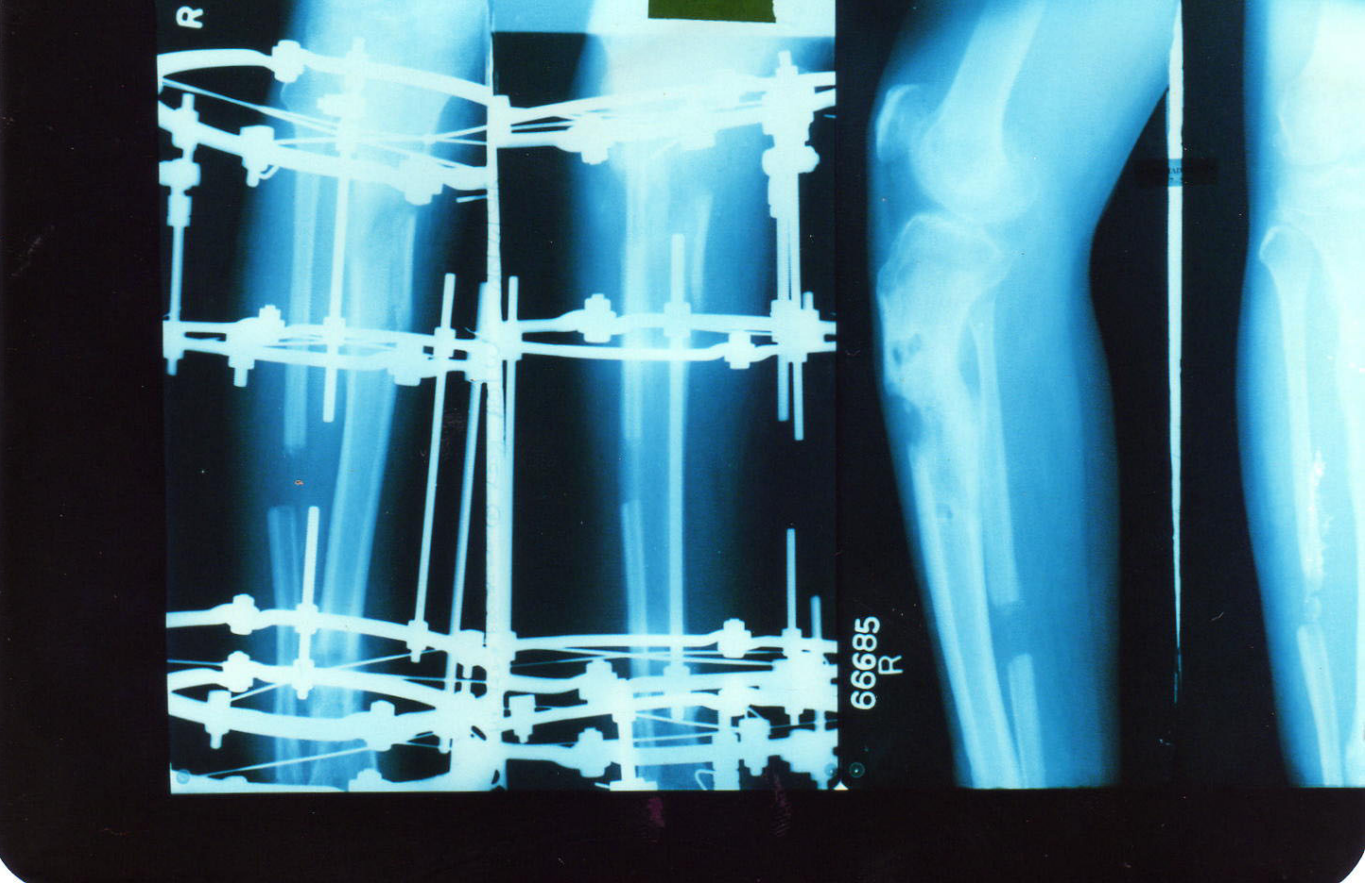
Patient no 11: Follow up x ray at 8 months and after fixator removal.

**Figure 4:**
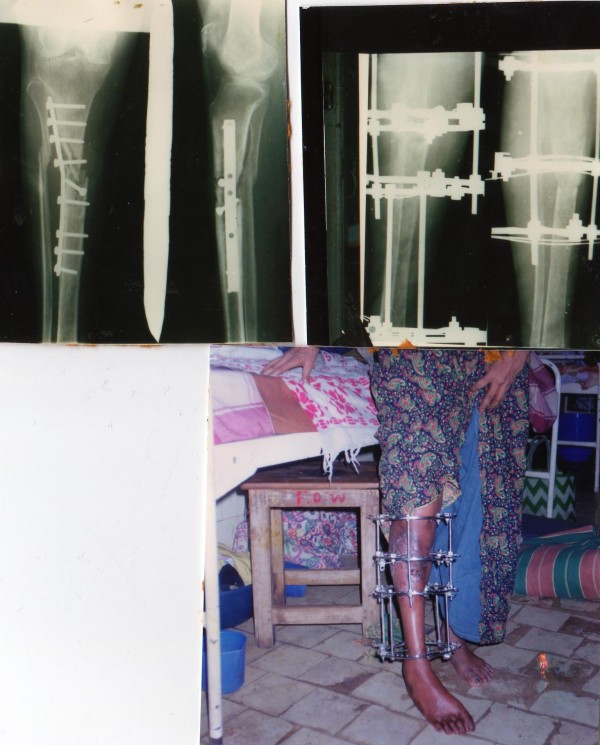
**Patient no 3: (clockwise from top left).  Pre-operative x-ray of the tibia; with fixator in situ at 100 days: and at 6 months.**

**Figure 5:**
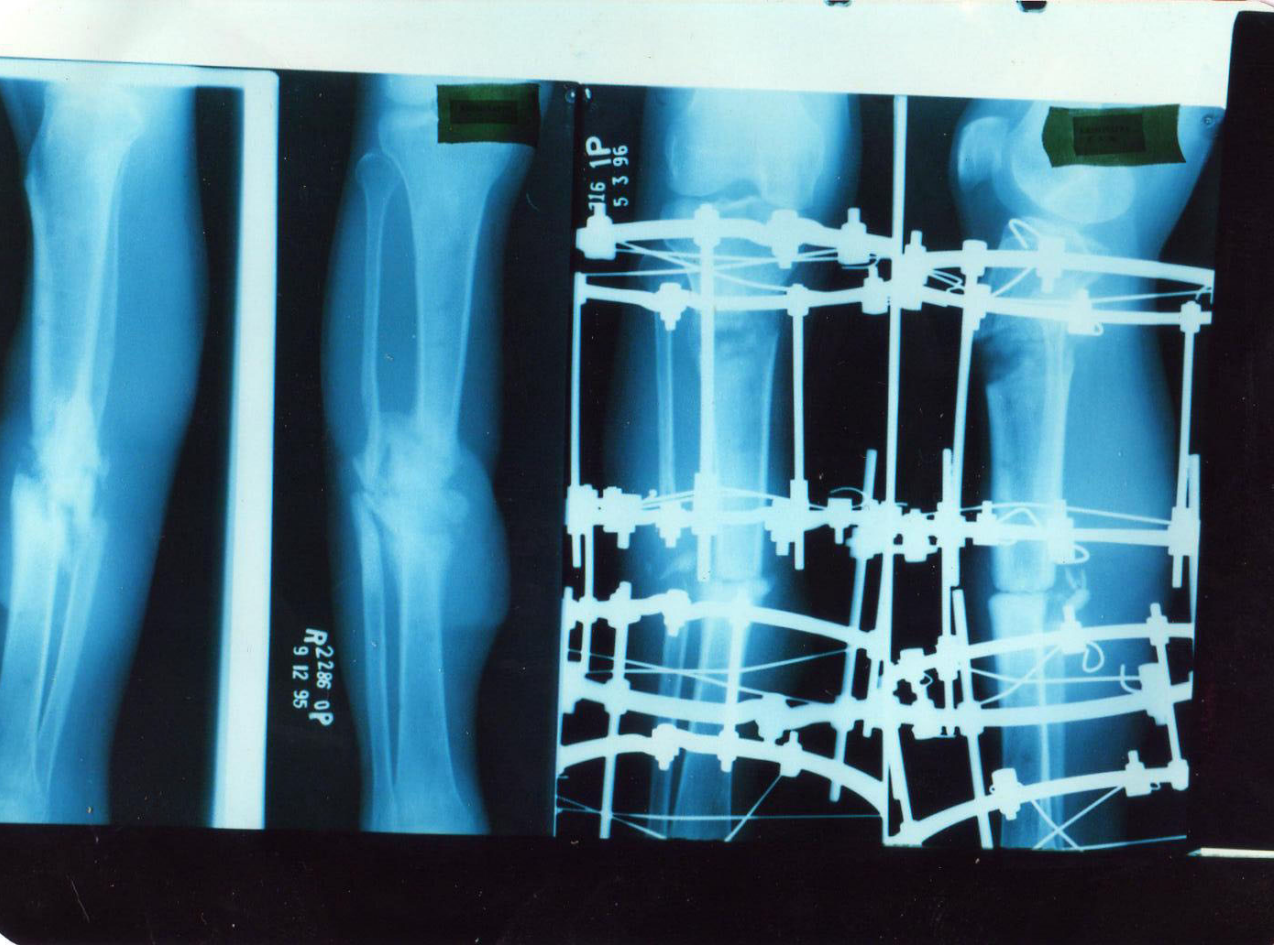
Patient no 18: Pre-operative x-rays; and at 6 months follow up with the fixator in situ.

The demographic details of patients are as per additional file 1. The study group consisted of 22 patients in the age group of 20 – 52 years [Average: 37.2]. There were 18 male and 4 female patients. All patients had limb oedema, equinus deformity of the ankle [12–23 degrees, Average: 15.7 degrees], ankle, and sub-talar and knee joint stiffness [figures 1a, 2a, 2b, 3a, 3b, 4a, 4b, 4c, 5a, 5b]. Fracture union was achieved in 18 patients without the need for bone grafting. The problems and complications in the cohort of patients studied are as per Table 1.

**Table 1:** Problems, obstacles and true complications in our cohort of patients

**Problems**		**Number of patients**
	Poor regenerate	2
	Delayed appearance of regenerate	12
	Pin tract infection	All
**Obstacles**		
	Infection needing change of frame/Wire	4
	Wire breakage	7
	Re-fracture	1
**True complications**		
	Chronic Osteomyelitis	*6
	Septic arthritis	2
	Persistent infection	4

* All patients had clinical and radiological evidence of Osteomyelitis at the time of initial presentation. 6 patients had persistent bone infection despite fracture union at final analysis.

In 1 patient, there was re- fracture and refused a re-application of the fixator. 3 patients discarded the fixator while on treatment. There were 167 episodes of pin-tract infection in all and 2 patients had abscess, cellulitis and septic arthritis of the knee, which required arthrotomy and drainage, change of wires and repositioning of the rings. Wire breakage was seen in 7 patients. Persistent bone infection despite bony union was evident in 6 patients. 4 patients had residual shortening of 3 cms and treatment was discontinued on patient's request after bone union was confirmed. There was limb oedema in all patients despite fracture union. Healing of sinuses and open wounds was satisfactory without the need for additional plastic surgical procedures. Knee, ankle and sub-talar joint stiffness persisted in all and worsened in 7 patients. Deformity correction in the sagittal plane was less satisfactory compared to the correction in the coronal plane. Bony and functional results [Tables 2 and 3] were evaluated as laid down by the ASAMI Criteria.

**Table 2:** Bony results in our patients

**Bone results**	**Grade**	**Criteria**	**Number of patients**
	Excellent	Union, no infection, Deformity < 7 deg, Limb length discrepancy < 2.5 cm	5
	Good	Union + any two of the following; absence of infection, < 7 deg deformity and limb length discrepancy of, 2.5 cm	8
	Fair	Union + one of the following;Absence of infection, < 7 deg deformity and limb length discrepancy of, 2.5 cm	5
	Poor	Non union/refracture/union + infection + deformity . 7 deg + limb length discrepancy . 2.5 cm	4

**Table 3:** Functional results in our patients

**Functional results**	**Grade**	**Criteria**	**Number of patients**
	Excellent	Active, no limp, minimum stiffness [Loss < 15 deg knee extension/, 15 degrees dorsiflexion of ankle], No reflex sympathetic dystrophy [RSD], insignificant pain	1
	Good	Active with one or two of the following:limp, stiffness, RSD, significant pain	4
	Fair	Active with three or all of the Following; limp, stiffness, RSD, significant pain	6
	Poor	Inactive [Unemployment or inability to return to daily activities due to injury]	*7 (10)
	Failures	Amputation	0

*The number would be 10 if all patients who lost or changed their occupation were to be considered as poor. 1 patient could not be contacted for a final functional analysis.

Of 22 patients in the study, 13 patients who underwent external bone transport, had an average bone gap of 4 cms [2–11 cms] with an average duration of fixation of 9.3 months [6.5–13 months]. There were 4 excellent, 3 good, 4 fair and 2 poor functional results and 1 excellent, 3 good, 6 fair and 2 poor results. 1 patient was lost for follow up at final functional analysis. 9 patients who underwent internal bone transport had an average bone gap of 5.4 cms [1.5–9 cms] with an average duration of fixation of 8.5 months [4–11 months]. There were 3 good, 4 fair and 2 poor bony results and 1 good, 3 fair, and 2 poor functional results.

3 Patients who rejected the fixator while on treatment and one patient who had a re-fracture after successful bony union were considered as poor results and therefore the functional results were considered as poor. One patient who had fair results in terms of bony union could not be contacted. Therefore functional result analysis was possible in 21 patients only.

## Discussion

In a developing country like India, the majority of the working population is served by public transport. The long distances required to travel for work place compounded by critical transport demands, increases the risk of road traffic accidents. These patients are often young, active and bread earners for their families.

The hospitals where the study was conducted are tertiary referral centres. Treatment cost is subsidised because of government funding for a vast majority of inpatient services. As in other studies [3], the rings and a few other components were recycled between patients, which reduced the financial burden to a large extent. However repeated hospitalisations, procuring expensive antibiotics not normally covered by the government services, regular follow ups in the fracture clinics, transport demands, imposed additional financial demands on the patients which were difficult to manage. Indirectly it also meant loss of working hours and thus a financial loss for the period of absence. These mostly in combinations led to rejection of the fixator during the course of treatment.

A fracture non-union is a significant problem to the patient and the surgeon. In many instances the patient has undergone one or more surgical procedures, has lost considerable time from his job or her life style, and has been forced to alter his or her life style. Furthermore, the psychological and physical trauma to the patient when faced with the prospect of another surgery is often underestimated. The problems facing the surgeon are no less formidable. In many instances consolidation of the non-union must be achieved with correction of axial and rotational mal-alignment [4]. In our study, most patients with open fractures were being treated with other forms of definitive treatment before they were referred for ring fixation. The previous multiple failed attempts at bone union, had exhausted all the financial resources and the patients were frustrated and depressed. It has been suggested that a referral to a specialised centre be made by at-least 6 months [5]. We propose a referral even earlier than the above suggested time duration of 6 months in open fractures in developing countries. This would aid better patient compliance and reduce the duration and cost of treatment.

In our study all patients had clinical and radiological features of chronic osteomyelitis by the time they were referred for definitive treatment. The mixed organism growth from bacterial cultures of nosocomial origin required repeated hospitalisations and expensive antibiotics for infection control. Despite being advised about proper pin site care, very few patients strictly adhered to the instructions. Two patients with septic arthritis of the knee who required arthrotomy and drainage were not co-operative and this directly correlated with the educational status of the patient. The pre-existing bone infection and insufficient pin care probably account for the higher incidence of pin tract infections in our study. We feel these patients are monitored under close medical supervision for optimal results.

The effects of smoking on the outcome of ring fixation have been well documented [6]. Majority of the patients in our study were smokers and consumed alcohol. Despite being advised about the consequences of smoking and alcohol intake, it was difficult to ensure complete co-operation from the patients in this regard. The regenerate appearance was not on expected lines particularly with the distal corticotomies. In one particular case, there was no regenerate visible on the radiographs even at 4 weeks and an ultrasound assessment was performed for confirming the same. An accordion man oeuvre was resorted to and distraction rate was reduced until satisfactory regenerate was visible.

The co morbidities in 5 patients did not appear to affect the final outcome in terms of bone and functional result. Other studies [7] have suggested multidisciplinary approach in the management of these patients. A vast majority of patients during the course of treatment were depressed and had to be counseled repeatedly. The reasons for depression were mainly due to the multiple failed attempts at union, pain, and financial and social constraints. Despite adequate counseling 3 patients discontinued the treatment and opted for amputation.

We have followed the criteria laid down by ASAMI. Other studies focus on the increased number of excellent bone results [3,5,7]. These studies have dealt with infected non- unions of long bones in general and not specifically to the Tibia. Treatment time with Ilizarov is lengthy with a considerable risk of complications [8]. Bone grafting at the docking site is recommended in order to shorten the duration of treatment and to prevent re-fracture and non-union. [9]. No patient required bone grafting in our cohort of patients.

Though acute docking followed by bone transport has been recommended for large tibial defects [10], our observations indicate that patients tolerate docking well upto 5 cms of shortening and the bony and functional results were uniformly poor beyond 5 cms. However, it may help shorten the duration of treatment and thus ensuring compliance.

The functional result is predetermined by the condition of the nerves, muscles, vessels, joints, and to a lesser extent the bone [11]. Ankle pain with disability is the major source of residual disability after successful use of the Ilizarov device for the treatment of Tibial nonunion even after fracture union [12]. No patient in our study had neurovascular deficits but the correlation between bony and functional results was poor. This is largely due to the soft tissue status particularly oedema and joint stiffness. In our study, all patients had varying degrees of knee, ankle and sub-talar joint stiffness. Though knee stiffness was largely overcome with physiotherapy, foot and ankle stiffness persisted and worsened despite bony union. This may account for the poor functional outcome in our cohort of patients.

ASAMI criteria define unemployment as a poor result. Majority of patients who were assessed for functional results did not go back to their original employment. Most changed their jobs to a sedentary and less demanding work as they did not have any choice. Other studies [5,7] have highlighted that patient satisfaction is more important than employment status in assessment of functional status. This is true in developed countries, where there is adequate government support for economic inactivity. In developing countries like India no such support exists. Therefore the direct applicability of the ASAMI criteria in the Indian scenario may not be appropriate for a finite functional analysis. Though many from our study were happy in that an amputation was avoided, most of them felt that this was at a 'heavy price' and some still preferred an amputation in the hope of early return to work and pain relief.

In our study, all patients' belonged to the middle and lower economical status of the society. We agree with other studies, which have highlighted the importance of economical support and patient motivation in the final outcome of limb salvage [13]. As in other studies [3,13], patient motivation, social and economical support is crucial in the final outcome of treatment. In our study patients with optimal outcomes had a working knowledge of the fixator probably because of their educational background. In developing countries, we believe patient satisfaction is dependant to a large extent on the social and economical situation. It is important to discuss the expected outcomes in terms of the duration of treatment, functional recovery and financial implications before hand.

Our observations indicate that the Ilizarov method is not a panacea but an important treatment method for surgeons, in situations with no good alternatives, such as osteomyelitis, osteopenia, complex deformities and significant limb-length inequalities. The drawbacks of this method are the time and resource, intensive nature of the treatment, the difficulties of prolonged fixator use and the potential major and minor complications. The surgeon should know when to offer an amputation as this is, in certain circumstances, the best option. Therefore the treatment in these situations needs to be highly individualised.

## Conclusion

Treatment of infected non-unions of tibia with Ilizarov ring fixation is effective but for optimal functional results the treatment needs to be highly individualised in developing countries. Early transfer to a specialised unit, patient selection and education regarding the duration of treatment, emotional, financial and social support are absolutely essential.

## Competing interests

The authors declare that they have no competing interests.

## Authors' contributions

TRM is the principal author of this manuscript and was involved in organising the study, collection of data, arranging patient care, analysis of results, and preparing the manuscript. BR was involved in collection of literature and proof read the manuscript. KSM, HMS, DCS, NK were responsible for overall patient care and decision-making. DCS and NK were involved in the analysis, interpretation and proof reading of the manuscript. All authors read and approved the manuscript.

## Supplementary Material

Additional file 1 Additional Table 1. Demographic data of patient Cohort Click here for file
